# Comparing continent ileostomy (CI) conversion to repair/redo IPAA: favorable outcomes

**DOI:** 10.1007/s00384-023-04555-x

**Published:** 2023-10-31

**Authors:** Nils Karl Josef Ecker, Christian Dinh, Gabriela Möslein, Karl-Wilhelm Ecker

**Affiliations:** 1 Forstmeisterweg 65, D-23568 Lübeck, Germany; 2 Iburger Straße 116, D-49082 Osnabrück, Germany; 3 Center for Hereditary Tumors, Ev. Krankenhaus BETHESDA, University of Düsseldorf, Heerstraße 219, D-47053 Duisburg, Germany; 4 Dept. of General, Vascular, and Pediatric Surgery, University of Saarland, Homburg, Saar, Germany; 5 Surgical Dept., MediClin Müritz-Klinikum, Weinbergstraße 19, D-17192 Waren, Germany; 6 Tannenweg 1, D-22889 Tangstedt, Germany

**Keywords:** Ileoanal pouch, Pouch failure, Continent ileostomy, Redo ileoanal pouch, Pouch survival, Patient’s satisfaction

## Abstract

**Purpose:**

This study aims to compare the outcomes of repair/redo ileal pouch-anal anastomosis (repair/redo-IPAA) with the conversion of IPAA to continent ileostomy (CI) in an effort to prevent the need for a permanent ileostomy (IS) following IPAA failure.

**Methods:**

This research involved a retrospective analysis of surgical records, employing descriptive statistics and Kaplan-Meier survival analysis.

**Results:**

Among 57 patients with an IPAA, up to three revisions were necessary due to complications or complete failure. Ultimately, repair/redo-IPAA preserved the IPAA in 14 patients (24.6%), conversion to CI salvaged the pouch in 21 patients (36.8%), and IS was unavoidable in 22 patients (38.6%). The cumulative probability of requiring conversion surgery was calculated to be 54.0% at 20 years, thereby reducing the cumulative risk of IS to 32.3%. The 20-year cumulative probability of pouch salvage by repair/redo IPAA was only 21.9%. However, this rate increased to 67.7% when conversion procedures were considered. Following repair/redo-IPAA, only 8.3% of patients reported evacuation frequencies of ≤ 4 during the day, and 16.7% were evacuation-free at night. In contrast, after conversion to CI, 98.0% of patients reported a maximum of four evacuations in a 24-h period. After undergoing repair/redo IPAA, between half and two-thirds of patients reported experiencing incontinence or soiling, while complete continence was achieved in all patients following conversion to CI. Notably, the majority of patients expressed overall satisfaction with their respective procedures. A positive correlation was identified between very high subjective satisfaction and positive objective surgical outcomes exclusively in patients who underwent conversion to CI.

**Conclusion:**

When complications or failure of IPAA occur, conversion to CI emerges as a highly viable alternative to repair/redo IPAA. This conclusion is supported by the observation that patient satisfaction appears to be closely tied to stable surgical outcomes. To reinforce these findings, further prospective studies are warranted.

## Introduction

For several decades, ileum-pouch-anal anastomosis (IPAA) has been the preferred reconstructive procedure in proctocolectomy [1]. However, ongoing developments in the field indicate the need for further advancements. While perioperative morbidity and long-term functional complications remain acceptable at experienced “high-volume centers” [2], the growing number of patients undergoing IPAA surgery has led to an increase in late complications, including complete pouch failure. Consequently, the long-term prognosis of IPAA, even in the hands of skilled surgeons, is not definitively predictable [3, 4].

Given these circumstances, revisional surgery holds significant importance. While minor functional complications may be resolved through simple repair surgery, complex complications and pouch failure often require redo surgery. Furthermore, in selected cases of Crohn’s disease, the possibility of avoiding permanent ileostomy (IS) is no longer excluded [5, 6]. Redo surgery typically involves redoing the anastomosis or performing partial or complete pouch reconstruction [7, 8]. Another option is conversion surgery, where the existing ileum pouch is converted into a continent ileostomy (CI) [9, 10]. In all approaches, the primary objective is to preserve the pouch safely. However, there is currently no rigorous evidence to determine the most promising approach for each specific condition [11].

Our extensive practice over nearly four decades has involved intensive work with both IPAA and CI [12–14]. Consequently, patients experiencing complications or failure of IPAA are often referred to our center for the decision-making process regarding repair or redo surgery versus conversion to CI. In addition to our own IPAA patients with functional complications, we have gathered a diverse cohort of IPAA patients with complications of varying localizations and severity. This offers us a unique opportunity and challenge to comprehensively investigate and evaluate the complications associated with IPAA and the available treatment options.

## Patients and methods

### Study design

This study presents a retrospective analysis of medical records of patients who underwent at least one revisional surgery for long-term complications of ileum-pouch-anal anastomosis (IPAA). The analysis covers the period from 1986 to 2003 at the Surgical University Hospital in Homburg, Germany, and from 2003 to 2016 at the MediClin Müritz-Klinikum in Waren, Germany. Only patients who initially received IPAA and underwent revisional surgery were included in the study. A demand-oriented follow-up, supplemented by a telephone interview conducted by one of the authors (CD) in 2018, was offered to the patients.

Classification of revisional surgery:

- Type A: salvage of IPAA through local proctological or transanal repair or redo interventions.
- Type B: salvage of IPAA through abdominal repair or redo surgery.
- Type C: conversion of IPAA into continent ileostomy (CI).
- Type D: disconnection of IPAA with loop-ileostomy (LI) or sacrification of IPAA with terminal ileostomy (TI).

Surgical treatment policy:

- Primary goal: restore continence control.
- Secondary goal: preserve the pouch as a valuable reservoir without scarification of small bowel.Outcome measures:

Outcome measures:

1. Postoperative morbidity
2. Pouch survival rate

## Assessment of surgical outcome and function

Most parameters were measured in absolute and relative numbers. However, desirable optimums were defined as frequencies of events ≤ 4 during the day and 0 at night to facilitate comparisons between the final states of IPAA, CI, and IS. Overall satisfaction was evaluated for each procedure.

## Data collection and statistics

Data from patient records and telephone interviews were entered into a database using IBM™ SPSS statistics software. Descriptive statistics were calculated, and we employed Kaplan–Meier analyses through XLSTAT, an add-on for Excel, to estimate the likelihood of an event’s occurrence or non-occurrence within a specified time interval. Due to the limited number of cases in our study, we did not conduct tests for statistical significance of differences.

## Results

### Patients IPAA history

A total of 57 patients (27 males and 30 females) with an average age of 39.2 ± 11.9 years at their first revisional surgery were included in the study. Among them, 41 patients had inflammatory bowel disease (IBD), whereas 16 patients had non-inflammatory bowel disease (non-IBD). Primary IPAA construction was performed on average 3.7 ± 3.9 years prior to the study. Of these patients, 29 underwent the primary IPAA procedure at our institution, while 28 patients received primary IPAA by other surgeons. Among the latter group, 11 patients (39.3%) had already undergone at least one revisional procedure elsewhere (Table 1).

**Table 1:** Patients and IPAA history

			**Number**	**Percent**
**Sex**				
**Patients**			57	**100.0**
Male			− 27	− 47.4
Female			**− **30	− 52.6
**Underlying disease**				
**Inflammatory bowel disease (IBD)**			**41**	**71.9**
Ulcerative colitis (UC)			−35	
Crohn’s colitis (CC)			−6	
**Non-IBD**			**16**	**28.1**
Familiar adenomatous polyposis (FAP)			14	
Slow transit constipation (STC)			2	
**Previous IPAA construction**				
**Own institution**			**29**	**50.9**
**External institutions**			**28**	**49.1**
Thereof with previous revisions			− 11	
**Characteristics at first revision**				
		**M ± S D**	**Median (range)**	
**Age at first revision**	(years)	**39,2 ± 11,9**	**39.0 (18–63)**	
**Time since IPAA construction**	(years)	**3.7 ± 3.9**	**3.0 (0–15.0)**	
Own institution (KWE)		4.3 ± 4.6	3.0 (0–15.0)	
External institutions		3.0 ± 2.9	2.0 (0–10.0)	

**Fig. 1:**
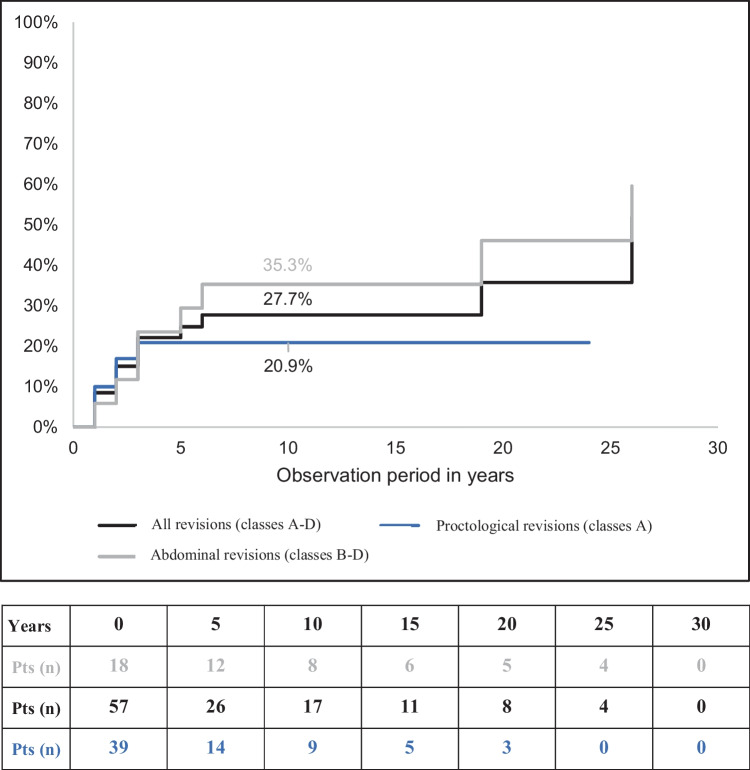
Cumulative probability of a second revisional surgery

During the treatment period, up to three revisional surgeries per patient were performed, covering procedures from all revisional types. One-third of the 73 total interventions belonged to type A, while the remaining two-thirds were spread across Types B–D. Proctological and abdominal revisions could occur as first or subsequent operations (Table 2). After the first revisional procedure, the overall probability of a second revisional procedure increased to 27.7% by the 6th postoperative year. The probability was notably higher for abdominal revisions at 35.3% compared to proctological revisions at 20.9% (Fig. 1).

**Table 2:** Classification of revisions and procedures

**Number and order of procedures**	**All**		**Class A (*n*)**	**Class B–D (*n*)**
	**(*n*)**	**%**		
**Number of revisions**				
One	43	75.4	10	33
Two	12	21.1	8	4
Three	2	3.5	-	2
**Total patients**	**57**	**100.0**	**18**	**39**
**Order of revisions**				
As first	57	78.1	18	39
As second	14	19.2	4	10
As third	2	2.7	-	2
**Total procedures**	**73**	**100.0**	**22**	**51**

Class A is proctological/transanal approach; Classes B–D are abdominal approaches

### Indications for revision and choice of procedure

Functional disorders, including incontinence in 30.1% (*n* = 22) and discharge disorders in 27.4% (*n* = 20) were the primary indications for revision. Septic complications, such as perianal or abdominal abscesses or fistulas, were the main indication in 20 cases (27.4%). Pouchitis was observed in 6 cases (8.2%), while anal pain and ulceration were rare indications (*n* = 3; 4.1%). Out of all the operations, 22 (30.1%) fell under Type A and were performed peri- and transanally using different methods. Type B operations were performed only 6 times (8.2%). In contrast, conversion surgery (Type C) was performed in 26 cases (35.6%). Finally, permanent ileostomies (TI, *n* = 13; LI, *n* = 6) were required in 19 cases (26.0%) (Table 3).

### Postoperative complications and management

In total, 30 complications occurred in 22 out of 57 patients, resulting in a complication rate of 38.6% (Table 4). No complications were recorded after proctological procedures (Type A). However, septic complications occurred in almost every third patient undergoing abdominal revisional surgeries. Conversion surgeries (Type C) were associated with CI-specific complications, with a total of 22 complications observed in 14 out of 26 patients (53.8%). Complications in Type B could be surgically resolved. In Type C, two fistulas between the pouch and the outlet duct remained resistant to repair, leading to the need for CI resection. This resulted in a secondary increase in ileostomy (IS) constructions to *n* = 21 and a corresponding decrease in CI to *n* = 24. Presacral chronic infections after Class C and D revisions typically constituted long-lasting problems.

**Table 3:** Indications for revision and choice of procedure; 73 revisional surgeries (first, *n* = 57; second, *n* = 14; third, *n* = 2) as related to the classes of revision surgery (A–D)

**Indication**	**n**	**%**	**Special procedure**	**Class (*n*)**			
				**A**	**B**	**C**	**D**
Abscess/fistula	20	27.4	Perianal drainage	3			
			Perianal fistula repair	1			
			IPAA redo/repair		5		
			IPAA diversion				6
			IPAA excision				3
			IPAA conversion			2	
Difficult discharge	20	27.4	Cutting middle bridge^a^	9			
			Bouginage^b^	5			
			IPAA conversion			6	
Incontinence	22	30.1	Post anal repair	1			
			IPAA redo/repair		1		
			IPAA conversion			16	
			IPAA diversion				2
			IPAA excision				2
Malign. transform	2	2.8	IPAA conversion			2	
Anal pain	1	1.3	Staple removal	1			
Perianal ulceration	2	2.8	Skin excision	2			
Pouchitis	6	8.2	IPAA diversion				4
			IPAA excision				2
**Total**	**73**	**100.0**		**22**	**6**	**26**	**19**

^a^In the case of hand suture of pouch and hand-sutured pouch-anal anastomosis

^b^In the case of a double stapler anastomosis

### Indications for revision and choice of procedure

Functional disorders, including incontinence in 30.1% (*n* = 22) and discharge disorders in 27.4% (*n* = 20) were the primary indications for revision. Septic complications, such as perianal or abdominal abscesses or fistulas, were the main indication in 20 cases (27.4%). Pouchitis was observed in 6 cases (8.2%), while anal pain and ulceration were rare indications (*n* = 3; 4.1%). Out of all the operations, 22 (30.1%) fell under Type A and were performed peri- and transanally using different methods. Type B operations were performed only 6 times (8.2%). In contrast, conversion surgery (Type C) was performed in 26 cases (35.6%). Finally, permanent ileostomies (TI, *n* = 13; LI, *n* = 6) were required in 19 cases (26.0%).

### Postoperative complications and management

In total, 30 complications occurred in 22 out of 57 patients, resulting in a complication rate of 38.6% (Table 3). No complications were recorded after proctological procedures (Type A). However, septic complications occurred in almost every third patient undergoing abdominal revisional surgeries. Conversion surgeries (Type C) were associated with CI-specific complications, with a total of 22 complications observed in 14 out of 26 patients (53.8%). Complications in Type B could be surgically resolved. In Type C, two fistulas between the pouch and the outlet duct remained resistant to repair, leading to the need for CI resection. This resulted in a secondary increase in ileostomy (IS) constructions to *n* = 21 and a corresponding decrease in CI to *n* = 24. Presacral chronic infections after Class C and D revisions typically constituted long-lasting problems.

**Table 4:** Postoperative major surgical complications as related to the types of revisional surgery (A–D)

**Type of complication**	**All** **(*n* = 73)**	**Class A** **(*n* = 22)**	**Class B** **(*n* = 6)**	**Class C** **(*n* = 26)**	**Class D** **(*n* = 19)**
	***n***	***n***	***n***	***n***	***n***
**Abdominal**					
Suture dehiscence (leaks)	**2**	n.a	0	2	0
Abscess/fistula	**7**	0^b^	2	3	2
NV instability	**5**	n.a	n.a	5^a^	n.a
Pouch-/NV-fistula	**6**	n.a	n.a	6	n.a
**Pelvic/presacral**					
Abscess/fistula	**10**	n.a	0	6	4
**Total**					
Complications (*n*)	**30**	0	2	22	6
Patients with compl. (*n*)	**22/57**	0/22	2/6	14/26	6/18
(%)	**38.6**	0.0	33.3	53.8	33.3

*n.a. *not applicable

^a^Nipple slippage/shortening (3), prolapse (2)

^b^Planned secondary healing is not considered a complication

### Preservation of pouch-anal continence

**Fig. 2:**
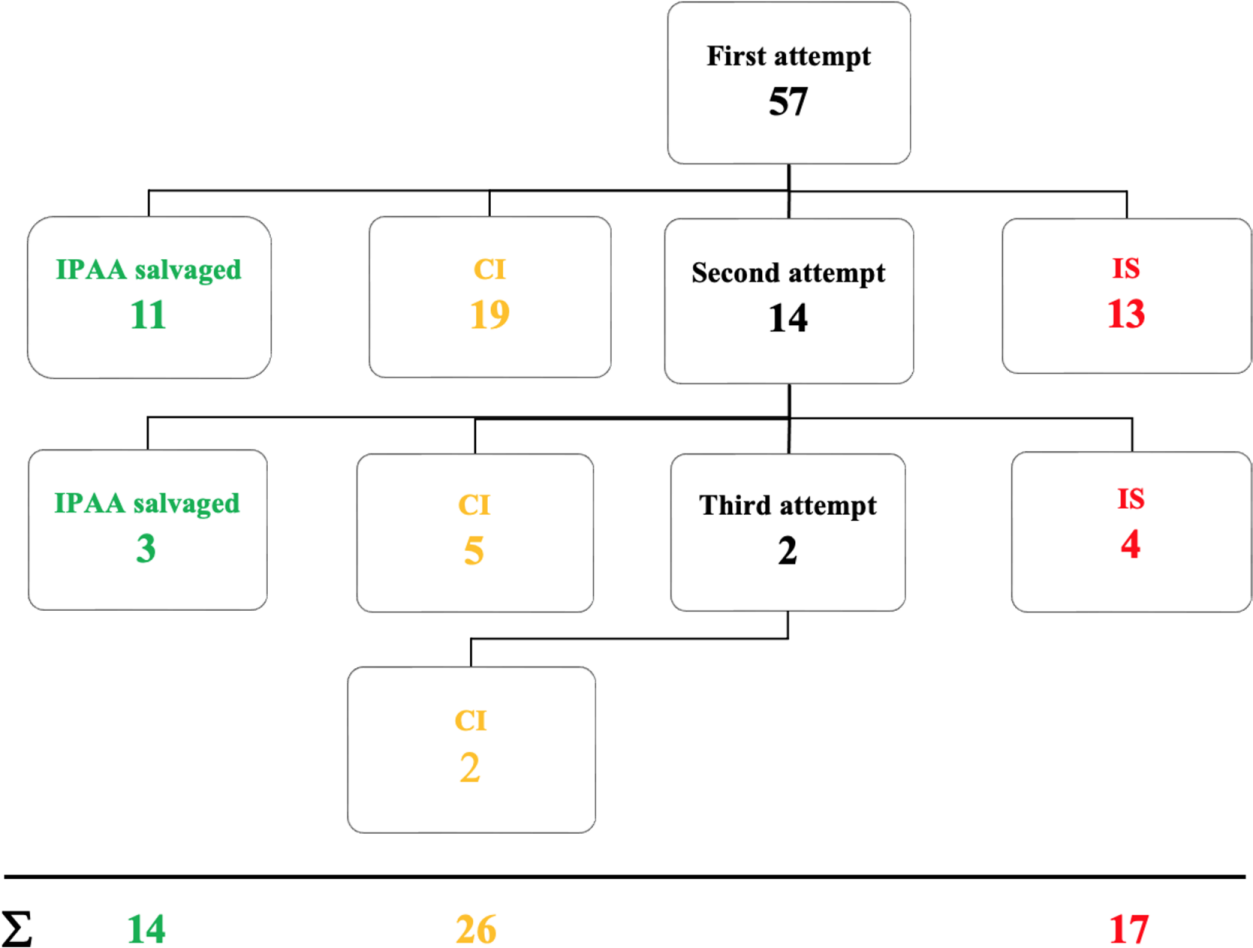
Flowchart, type, sequence and outcome of salvage procedures

**Fig. 3:**
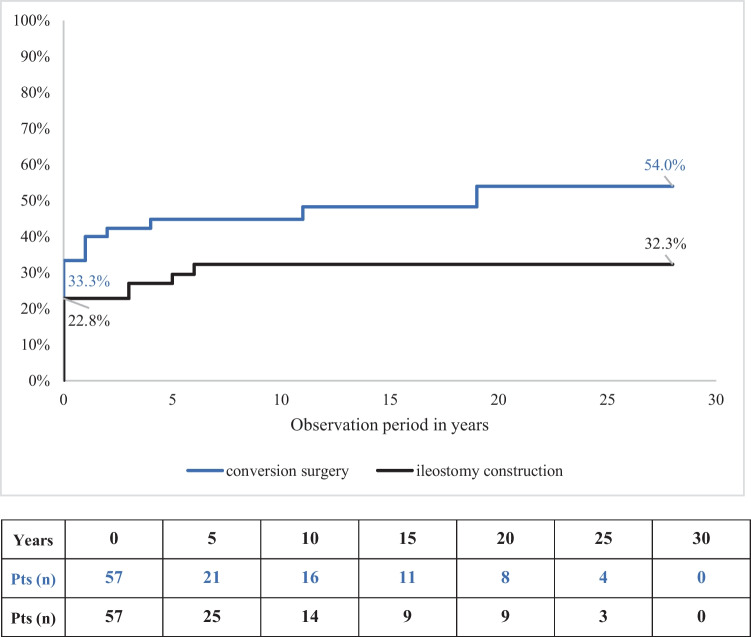
Cumulative loss of anal function: opportunity of conversion to CI versus risk of end ileostomy. Pts, patients with planned procedures under observation (changes due to complications are not considered)

During the first revisional surgery, pouch-anal continence could be preserved in 25 patients (43.9%). Out of these, only 11 patients (44.0% or 19.3% of the total collective) maintained stable continence until the end of the observation period, while the remaining 14 patients required at least one additional revisional surgery. In 32 patients (56.1%), anal continence could not be maintained. Among them, 13 patients (40.6% or 22.8% of the total collective) required IS (LI or TI). However, conversion of IPAA to CI successfully restored continence in 19 patients (59.4% or 33.3% of the total collective). During the second and third revisional surgeries, IPAA could be salvaged in only three out of 14 patients (21.4%), while four additional patients (28.6%) had to accept IS. Successful conversion to CI was possible in seven patients (Fig. 2). Overall, pouch-anal continence was successfully maintained in only 14 out of 57 patients (24.6%). The risk of losing pouch-anal continence in favor of IS or CI was calculated at 22.8% and 33.3%, respectively, for the first revisional surgery. The risk for IS remained constant at 32.5% from the 6th postoperative year onward, while the risk for CI increased to 54.0% by the 19th year (Fig. 3).

### Salvage of the pouch

**Fig. 4:**
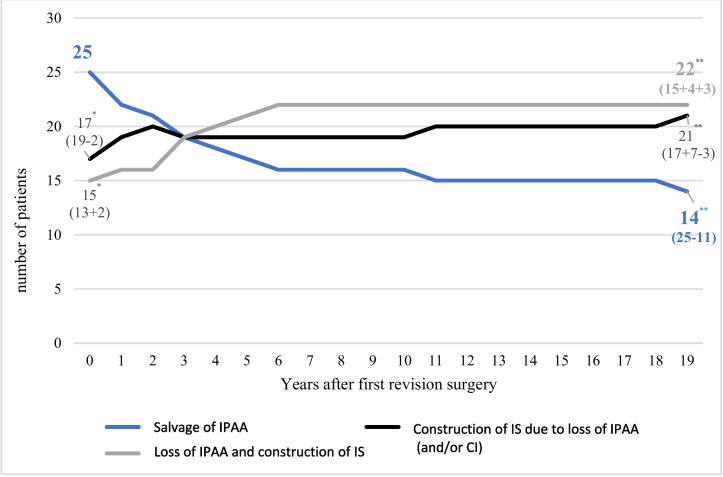
Fate of the IPAA after revisional surgery in the long-term follow-up. IPAA, ileum-pouch-anal anastomosis; CI, continent ileostomy; IS, ordinary ileostomy; *n*, number of patients under observation. *Realized procedures at the first revision surgery (intended procedures ± changes by postoperative complications). **Final procedures 19 years after the first revision surgery (initially realized procedures ± changes by long term complications)

**Fig. 5:**
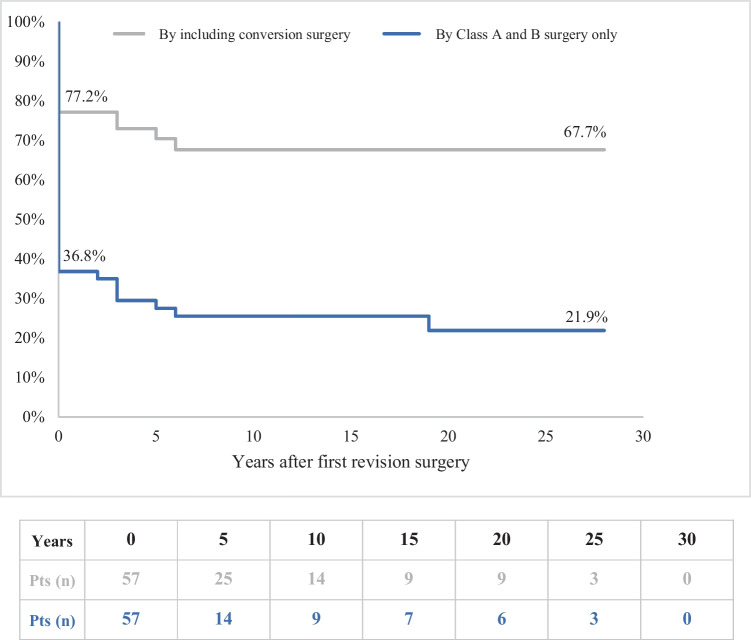
Cumulative probability of long-term revisional pouch survival with and without conversion surgery. Pts, patients under observation

With the first revisional surgery, the pouch could be salvaged in 25 patients (43.9%). In two out of 19 patients who underwent initial IPAA conversion, the pouch had to be sacrificed due to early complications. Consequently, only 17 patients (29.8%) entered long-term CI follow-up, and 15 (26.3%) had IS. Nineteen years later, after the second and third revisional surgeries, only 14 (56.0%) of the previously maintained IPAAs were still functional, corresponding to 24.6% of the total collective. Among them, seven had been converted to CI, while four were resected or excluded with TI or LI. Due to the loss of three CIs due to long-term complications, the total number of CIs was only 21, and the number of IS reached 22 (Fig. 4). The probability of pouch survival through redo-IPAA surgery was calculated at 36.8% for the first revisional and dropped to 21.9% by year 19. In comparison, the probability of pouch survival through both redo-IPAA and conversion to CI was calculated to be 77.2% at the first revision and 67.7% at postoperative year 6, remaining constant thereafter. Thus, the probability of pouch survival was 2–3 times higher over time when pouch conversion was also employed (Figs. 4 and 5).

### Final surgical outcomes, function, and satisfaction

After redo IPAA, 8.3% of patients achieved an optimal level of ≤ 4 defecations during the day, while 16.7% were defecation-free at night. In contrast, almost all patients (98.0%) who underwent conversion to CI managed with ≤ 4 intubations during a 24-h period. Among IS patients (LI and TI), only approximately 50% managed with ≤ 4 ostomy care sessions per 24 h. Reliable anal continence was reported by 41.7% of patients after redo IPAA during the day and by one in four during the night. By contrast, after conversion to CI, all patients reported complete competence of the nipple valve. Perfect stomas were more common in TI than in LI (83% vs. 40%). Half of the patients who underwent redo-IPAA reported leakage or soiling during the day, and two-thirds experienced this inconvenience during the night. The proportion of patients forced to wear incontinence pads was as high as 75–90%. Similar, albeit less dramatic, reports were made by patients after LI due to mucous secretions. In cases where the anal continence was sacrificed, as in conversion and TI, persistent perineal wound secretions were present in one-quarter to one-third of all cases, requiring the use of absorbent dressings. Despite these circumstances, overall a surprisingly high proportion of patients (78.6%–98.0%) expressed their satisfaction, irrespective of the specific procedure and despite different comorbidities.

## Discussion

In ileal pouch-anal anastomosis (IPAA), the observed early and late morbidity contrasts significantly with functional outcomes, which are generally assessed as excellent, accompanied by high patient satisfaction rates [4, 15]. This juxtaposition is noteworthy and warrants further investigation. Notable causes of morbidity include septic pelvic complications (23.0–54.5%), compromised or lost continence (12.0–44.6%), and inflammatory recurrence of the underlying disease (10.0–25.0%) [7, 11, 16–18]. Our data align with these findings, substantiating a broad range of indications for repeated surgical interventions. These interventions can result in either the salvage or the loss of the pouch. Subsequent to these interventions, new pouch failure rates of 20%–40% are reported, necessitating further revisions [6, 8, 19]. We have observed this trend to apply increasingly to both abdominal (classes B-D revisions) and, to a greater extent, proctological revisions (type A). Therefore, revision surgery emerges as a pivotal component in the long-term strategy for sustaining functional outcomes and, consequently, patient quality of life (QoL) [8, 20].

In existing literature, various corrective surgeries following IPAA are depicted in isolation. Contrarily, in this study, we classified established revision surgeries into four distinct categories (A–D), enabling more refined comparisons of procedures (both in terms of initial goals and subsequent outcomes).

### Redo versus conversion procedures

After redo IPAA, Theodoropoulos et al. reported an “overall” morbidity rate of 41.4% due to pouch fistulae, strictures, pelvic abscesses, pouchitis, wound infections, small bowel obstruction, and pouch-vaginal fistulae. This was based on a systematic review and meta-analysis of 77 studies involving a total of 2103 patients [11]. The rate of follow-up revision was calculated at 27.1%, and the definitive failure rate stood at 19.0%. Similar findings have been reported from Cleveland, OH/USA [5]. Regarding conversion to CI surgery, a review of 8 publications with 143 patients identified a postoperative morbidity of 35.2%, a revision surgery rate of 44.1%, and a long-term success rate of 88.8%, corresponding to a failure rate of 11.2% [14].

In our study, the repair/redo category (comprising type A and B revisions) accounted for 28 out of 73 procedures (38.4%). Although the complication spectrum in our cohort is comparable to literature findings, the long-term success rate of 56.0% (*n* = 14/25) after 19 years is notably lower than the 71.9% cited in the aforementioned meta-analysis [11]. We interpret this discrepancy not as a reflection of inferior surgical outcomes per se, but rather as a divergence in procedural selection from the prevailing norms, or the “mainstream”. Our objective for revision surgery was to achieve the best possible functional outcome at each time point. Accordingly, externally failed redo IPAAs were systematically excluded from repeat redo procedures, favoring conversion instead. Moreover, the criteria for IPAA conversion to continent ileostomy (CI) were deliberately liberalized, particularly when preoperative assessments raised concerns regarding the prospective success of a redo IPAA, or when the outcome of a redo IPAA was deemed suboptimal (second goal). As a result, the numbers of repair/redo and conversion procedures in our cohort were virtually equal (*n* = 28 and *n* = 26, respectively).

In our findings, the primary risk factor for ileostomy is incontinence, closely followed by various forms of continence disturbances, including difficult defecation. While septic and inflammatory pouch-anal complications, as well as malignant transformations, are less common risk factors (as detailed in Table 3), they are still noteworthy. Given these risk factors, we recommend considering conversion surgery over redo surgery. This approach offers the best opportunity to circumvent the challenges of incontinent ileostomy.

**Table 5:** Overall functional and surgical outcome

**Outcome** **parameters**	**IPAA salvaged by repair/redo IPAA (*n* = 14)**		**IPAA converted into CI (*n* = 21)**		**LI (IPAA left in situ) (*n* = 7)**		**TI (IPAA resected) (*n* = 15)**	
	***n*/*n***^**a**^	*** %***	***n*/*n***^**a**^	***%***	***n*/*n***^**a**^	***%***	***n*/*n***^**a**^	**%**
**Frequencies**	***Normal defecation***		***Intubation of abdom. reservoir***		***Change of ileostomy bag***		***Change of ileostomy bag***	
Day time ≤ 4	1/12	8.3	20/21	98.0	2/5	40.0	6/12	50.0
Night time 0	2/12	16.7			3/5	60.0	6/12	50.0
**Full function**	***Anal continence***		***nv competence***		***Perfect stoma***		***Perfect stoma***	
Day time	5/12	41.7	21/21	100.0	2/5	40.0	10/12	83.3
Night time	3/12	25.0						
**Anal/perineal deficiencies**	***Leakage/soiling***		***Wound secretion***		***Mucous discharge***		***Wound secretion***	
Day time	6/12	50.0	5/20	25.0	3/5	60.0	3/10	33.3
Night time	8/12	66.7						
**Need for anal or perineal care**	***Incontinence pads***		**Wound dressing**		***Incontinence pads***		**Wound dressing**	
Day time	9/12	75.0	5/20	25.0	3/5	60.0	3/10	33.3
Night time	10/12	91.7						
**Overall satisfaction**								
	**11/14**	**78.6**	**20/21**	**98.0**	**4/5**	**80.0**	**10/12**	**83.3**

*IPAA* ileal-pouchanal anastomosis, *CI* continent ileostomy, *LI* loop ileostomy, *TI* terminal ileostomy

^a^Patients with data

It is acknowledged that repeated redo IPAA procedures are correlated with diminishing pouch survival [8] and successive deterioration of function with each intervention [21]. In contrast, Denoya et al. reported a 10-year CI pouch survival rate of 93.5% after revision of the nipple valve for instability in CI patients [22]. Accounting for inflammatory pouch complications, the cumulative pouch survival rate for CI remains robust, albeit decreasing to just under 80% after an extended duration of 44 years [23]. For conversion surgery, recent data suggests a revision rate of 21.7% and a 5-year pouch survival rate of 80.3% [14]. Collectively, these findings suggest that the surgical outcomes, both initially and subsequently, of conversion surgery may be more favorable than those associated with redo IPAA procedures.

### Anal continence versus pouch evacuation control

In the meta-analysis by Theodoropoulos et al., functional success following redo-IPAA was characterized by a mean of 6.5 ± 1.4 daytime defecations, 1.8 ± 1.82 nighttime defecations, and reported rates of daytime leakage at 21.9 (8.5–35.0)% and nighttime soiling at 38.4 (21.7–55.2)% [11]. Comparable results have been observed in Cleveland, OH, USA [6]. For enhanced comparability of functional outcomes in the present study, the precise limits and scales of Continent Ileostomy (CI) were employed as a “benchmark” for IPAA. While continence and defecation frequency in IPAA demonstrate a broad natural variation (expressed as means ± standard deviations), CI operates on a more binary principle — often termed the “all-or-nothing law.” This encompasses voluntary evacuation control, characterized by effortless intubation of the pouch, flawless competence of the nipple valve for liquid stool and gas, and evacuation frequencies of three to four times per 24 h at most [24, 25]. Inferior results are typically indicative of mechanical complications related to the nipple valve; these complications are consistently amenable to surgical correction [21, 22, 25]. Therefore, CI’s evacuation control offers a uniquely robust and unparalleled degree of functional reliability compared to often suboptimal anal continence in IPAA (Table 5).

### Incontinent versus continent ileostomy

In managing IPAA complications, ileostomy (IS) represents the final therapeutic recourse. Two pathways exist: either preservation of the pouch in situ with loop ileostomy (LI) or end ileostomy (TI), or pouch resection, which invariably culminates in TI. Despite heightened perioperative risks, pouch resection is favored by a majority of experienced surgical centers [18, 26]. Retaining the pouch in situ may lead to chronic pouch complications, problematic perianal mucus discharge, stoma care issues, and complications due to inadequate fecal diversion [27]. Conversely, post-resection healing disorders of the sacral cavity may develop [27]. Although this complication can also arise following conversion, the substantial benefits of CI—assured evacuation control and preservation of critical small bowel segments—overwhelmingly outweigh this drawback [14]. Given that IS also carries a significant risk of reoperation, the balance of advantages for patients clearly tips towards CI [28].

### Subjective patient satisfaction versus objective surgical outcome

Data from Cleveland reveals that over 90% of patients positively rate their surgery after redo IPAA. The authors infer that patients, driven by strong motivation, are inclined to accept these surgical outcomes, despite their imperfections [6]. Theodoropoulos et al. highlight high levels of patient satisfaction reported in pertinent studies [11]. Our investigation elucidates that overall patient satisfaction can be uniformly high across different procedures, without a direct correlation to surgical outcomes or function. This finding aligns with Theodoropoulos et al., who identified no correlation between functional outcomes and Quality of Life (QoL) in their meta-analysis following redo IPAA [11]. Thus, high motivation and satisfaction may more accurately reflect patients’ resilience and acceptance of their condition. It is imperative that surgical decision-making strives for a harmonious alignment between subjective patient satisfaction and objective surgical outcome, as this alignment was predominantly observed following conversion surgery in our study. We concur with R. Shuford and JH Ashburn that CI offers patients the best possible QoL and functional status post-proctocolectomy [29].

## Strengths and weaknesses

A notable strength of this study lies in its comprehensive comparative evaluation of all surgical procedures for addressing IPAA complications. However, the retrospective design and limited sample size constitute significant weaknesses.

## Summary and conclusions

Our study underscores that each established revisional procedure for IPAA harbors its unique advantages and drawbacks, and that risks for subsequent surgical interventions due to complications are inherent to all approaches. Sometimes, altering the surgical approach may prove more beneficial than repeating a previously performed procedure. The surgical burden imposed on the patient is an important consideration [15]. Accordingly, treatment strategies should strike a balance between addressing the complications inherent to revisional surgery (“first outcome measure”) and the surgery’s capacity to preserve or reinstate fecal control, however defined (“second outcome measure”). For patients, the competency of the surgeon in executing an alternative continence-restoring procedure, such as conversion to CI, is paramount. In comparison to redo IPAA, CI emerges as a superior option, as the high levels of patient satisfaction it garners are grounded in objectively optimal and reliable surgical outcomes. In the light of these results, the authors suggest that skilled pouch surgeons should be knowledgeable and gain experience with the different types of CI and under secured guidance (proctoring) establish the procedure at their institutions. Patients should be made aware of the different options including CI and be guided towards informed decision-making according to their own preferences and data available regarding outcomes. Future prospective controlled studies are advocated to further substantiate the procedural recommendations put forth in this study. An interesting additional topic for prospective publications will be to address and prospectively validate the four different types of CI. After abandoning the original K-Pouch [30], the authors exclusively perform the modified S-Pouch as a standard procedure for CI. However, additional techniques have evolved which are the T-pouch [31] and the BCIR (Barnett continent intestinal reservoir) [32]. Validation of these is a requirement for the future, especially taking the results of this study favoring CI versus redo IPAA into account.

## Data Availability

All original data are stored electronically by Christian Dinh. These are data from his dissertation. All data and materials used are secured digitally by the corresponding author.

## Code availability

No code available.
